# Combined Lead Extraction and Leadless VDD Pacemaker Implantation in d-TGA Patient After Senning Procedure

**DOI:** 10.1016/j.jaccas.2026.106866

**Published:** 2026-02-05

**Authors:** Hamza Hamayel, Fateh Awwad, Mohammed Abutaqa, Mohammed Abu Baker, Arab Ramadan, Yunis Daralammouri, Ibraheem Marie

**Affiliations:** a Department of Cardiology, An-Najah National University Hospital, Nablus, Palestine; b Department of Pediatric Cardiology, Palestinian Medical Complex Hospital, Ramallah, Palestine; c Department of Cardiac Surgery, An-Najah National University Hospital, Nablus, Palestine; d Department of Anesthesia, An-Najah National University Hospital, Nablus, Palestine; e Department of Medicine, Faculty of Medicine and Allied Medical Science, An-Najah National University, Nablus, Palestine

**Keywords:** atrioventricular block, congenital heart disease, lead extraction, leadless pacemaker, senning procedure

## Abstract

**Background:**

Conduction abnormalities are common long-term sequelae after the Senning procedure for dextro-transposition of the great arteries (d-TGA), often requiring permanent pacing. Infection or lead malfunction may necessitate system extraction. Leadless pacemakers offer a promising alternative option.

**Case Summary:**

We present a 30-year-old man with d-TGA who previously underwent a Senning procedure. He had an infected pacemaker with lead extrusion. A combined procedure was performed, including complete system extraction and implantation of a leadless VDD pacemaker (Micra AV2). The procedure achieved atrioventricular (AV) synchrony and rapid recovery.

**Discussion:**

This case highlights the viability of integrating leadless VDD pacemaker implantation with lead extraction in post-Senning d-TGA patients for efficient AV synchrony. It supports leadless pacing as practical solution for complex congenital cardiac anatomy.

**Take-Home Messages:**

Leadless pacemakers represent a promising alternative pacing strategy for patients with d-TGA post-Senning procedure. Micra VDD pacing with AV synchrony represents an innovative and effective approach in this setting.

## Graphical Abstract

**Figure:**
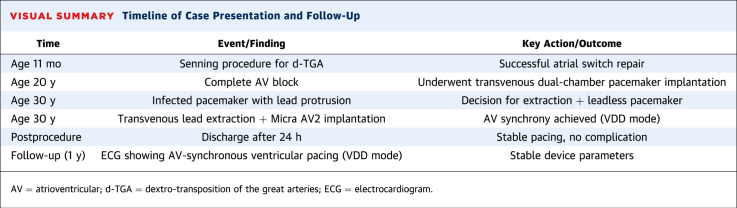

## History of Presentation

The patient was a 30-year-old man who had undergone a surgical correction of dextro-transposition of the great arteries (d-TGA) (Senning procedure) at the age of 11 months. He presented with erythema, swelling, and purulent discharge at the pacemaker site for the previous 2 months. He had no fever, chills, shortness of breath, fatigue, or weight loss. A local examination showed redness and tenderness, with protrusion of one of the leads from the skin.

**Take-Home Messages**

• Leadless pacemakers represent a promising alternative pacing strategy for patients with d-TGA post-Senning procedure.
• The Micra VDD device with AV synchrony represents an innovative and effective approach in this setting.

## Past Medical History

At the age of 20 years, the patient had a dual-chamber pacemaker implanted via the left subclavian vein owing to complete heart block. Thorough investigation at that time revealed normal coronaries on cardiac catheterization, a patent baffle on computed tomography angiography, and severe dilation with reduced function (ejection fraction: 43%) of the systemic ventricle (anatomically right ventricle) on echocardiography and cardiac magnetic resonance imaging.

## Differential Diagnosis

The differential diagnosis included localized pacemaker pocket infection, device-related endocarditis, and superficial cellulitis or abscess.

## Investigation

**Figure 1:**
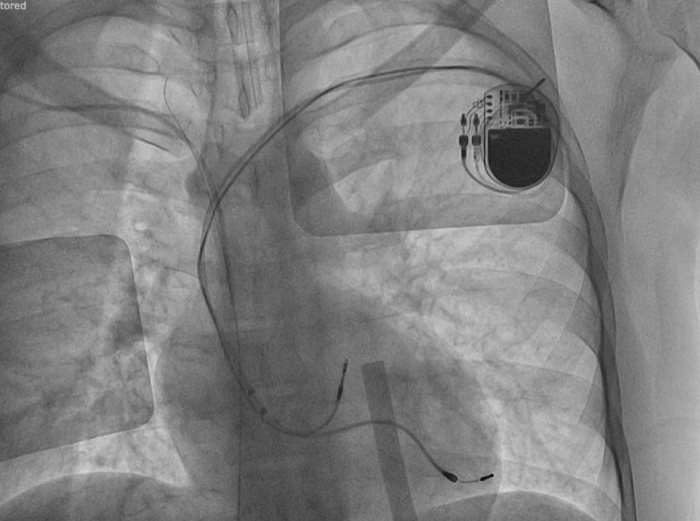
Anteroposterior Fluoroscopic View Showing Pacemaker Leads The atrial and ventricular leads were connected to the pacemaker, but the atrial pole of the VDD lead was not connected to the pacemaker and protruded through the skin.

Local examination revealed lead protrusion through the skin. Ultrasound showed signs of inflammation with no collection. Blood and fluid cultures were negative. Transesophageal echocardiography showed no signs of infective endocarditis. Fluoroscopy showed an atrial electrode in the left atrial appendage (within the subpulmonic atrium) connected to the pacemaker and a VDD lead in the anatomical left ventricular apex (subpulmonic ventricle). The ventricular part of the VDD lead was connected to the pacemaker, and the atrial part (pole) was not connected to the pacemaker but protruded through the skin (Figure 1). Interrogation of the device showed that the patient was in sinus rhythm but was pacemaker dependent owing to complete atrioventricular (AV) block.

## Management

**Figure 2:**
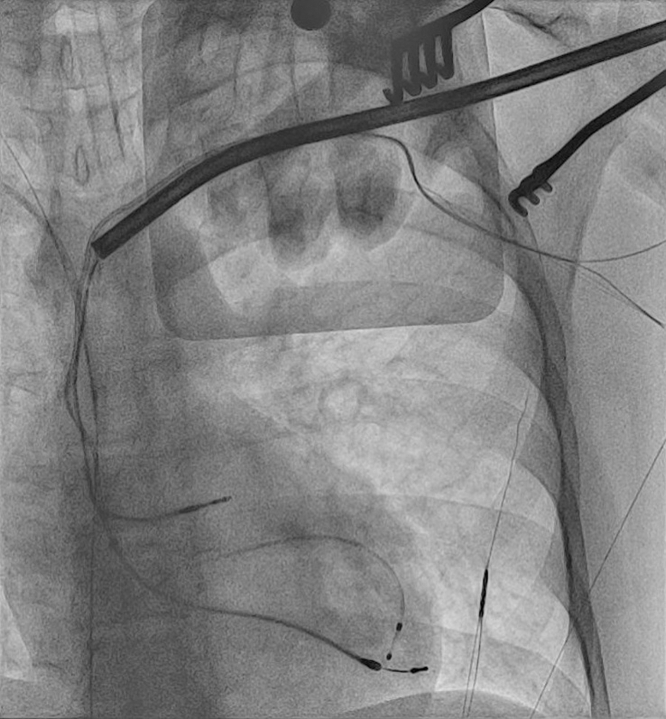
Anteroposterior Fluoroscopic View During Transvenous Lead Extraction The TightRail mechanical rotating sheath was advanced over the ventricular lead.

**Figure 3:**
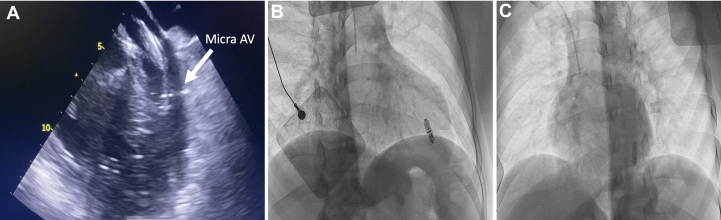
Imaging During and After Micra AV2 Leadless Pacemaker Insertion (A) Transesophageal echocardiography showing the delivery system of the leadless pacemaker crossing the mitral valve. (B and C) Fluoroscopic RAO 33° and LAO 30° views showing the Micra AV2 in the apical septum. LAO = left anterior oblique; RAO = right anterior oblique.

Extraction of the pacemaker system, including all leads, was recommended. Given the high risk for reinfection and the risk for baffle (after atrial switch surgery) stenosis and/or leak, insertion of a leadless pacemaker was decided upon after discussion with the patient. Informed consent was obtained from the patient. The procedure was performed under general anesthesia. A temporary transvenous pacemaker via the right femoral vein was placed before starting the procedure. The left-sided pocket (left infraclavicular region) was opened. Dissection was performed to uncover the sleeves of the atrial lead and the right ventricular VDD lead. Both leads were cut and secured with locking stylets (1 locking stylet in the atrial lead and 2 locking stylets in the right ventricular VDD lead), then both leads were completely extracted using a 13-F TightRail mechanical rotating sheath (Philips) (Figure 2). The infected skin and subcutaneous tissue had been debrided. Meanwhile, transesophageal echocardiography showed a patent baffle with no complications. After that, we proceeded with leadless pacemaker implantation. A dedicated long sheath was advanced to the inferior vena cava–baffle level via the right femoral vein. The leadless pacemaker delivery sheath was advanced via the dedicated sheath to the anatomical left ventricle (subpulmonic ventricle) crossing the baffle. The VDD leadless pacemaker (Micra AV2, Medtronic) was implanted at the apicoseptal position of the anatomical left ventricle, guided by transesophageal echocardiography and fluoroscopy (Figure 3, Videos 1 and 2). The delivery system and the long sheath were removed at the end of extraction, and the temporary pacemaker was also removed. The threshold was 0.7 V at 0.24 ms, impedance 700 Ω, and sensing amplitude 8.5 mV. The pacing mode was programmed in VDD mode with a lower rate of 50 beats/min and an upper tracking rate of 135 beats/min.

## Outcome and Follow-Up

**Figure 4:**
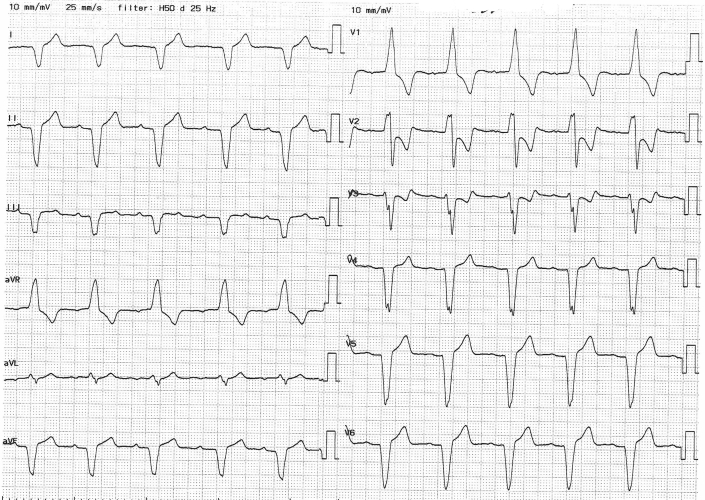
12-Lead Electrocardiogram After Insertion of Micra AV2 Leadless Pacemaker in VDD Mode Electrocardiogram shows sinus rhythm followed by synchronized ventricular pacing.

The patient did well with no complications, and he was discharged 1 day after the procedure. Electrocardiogram showed sinus rhythm and synchronized ventricular pacing (Figure 4). The electrical parameters of the device were stable for 1 year after implantation. The threshold was 0.5 V at 0.24 ms, impedance 830 Ω, and sensing amplitude 9.9 mV. Interrogation of the Micra device showed atrial mechanical sensing with ventricular pacing in 60% of the time. This relatively low AV-synchrony rate (<70%) is most likely due to sinus tachycardia during exercise.

## Discussion

This case report highlights the suitability and effectiveness of a combined procedure consisting of transvenous lead extraction and insertion of a Micra AV2 leadless pacemaker in a patient with d-TGA after a Senning procedure.

Bradyarrhythmia is a frequent long-term complication after the Senning procedure in d-TGA patients.^1^ Sinus node dysfunction makes up the majority (about 50% of cases), while about 9% of patients develop complete AV block.^2^ Around 30% of patients require pacemaker implantation.^1^ Epicardial pacemakers are prone to infection, lead fracture, and detachment.^3^ Currently, dual-chamber transvenous pacemakers with lead insertion are the standard treatment. They offer AV synchronization, making them more physiologic and more effective.^4^ Crossing the baffle to insert the atrial and ventricular leads is technically difficult. Additionally, device infection in these cases carries serious consequences related to baffle leak, stenosis, and thrombosis, which are difficult to treat. Transvenous lead extraction in case of infection or baffle failure is also technically difficult and challenging.^5^

Studies have shown that laser and mechanical lead extraction can be successfully performed after the Senning procedure in patients with d-TGA. The presence of a baffle makes lead extraction very difficult. Many patients may need baffle stenting after the procedure because of leak or stenosis, while in other reported cases the baffle remained patent and required no intervention.^5^ This highlights the importance of careful planning, including preprocedural imaging (such as transesophageal echocardiography in our case), in addition to being ready for baffle stenting when it is required.

Consideration of a leadless pacemaker is a promising option, as it has less risk of infection, and getting open superior vena cava access is not required. The use of leadless pacemakers after atrial switches in patients with d-TGA has been reported in 7 cases.^6^, ^7^, ^8^, ^9^, ^10^ Sinus node dysfunction was the indication in 5 patients, and AV block in 2 patients. Only 1 patient had dual-chamber leadless pacemaker (Aveir DR system, Abbott), which was upgraded after the initial Aveir VVI^6^; all other patients had Micra VR (Medtronic) implantation. Two patients underwent direct leadless pacemaker implantation without a trial of transvenous lead pacemaker implantation; the first patient had superior vena cava obstruction,^9^ and the second patient had no clear reason.^10^ In the remaining 5 patients, a leadless pacemaker was used after complications with epicardial leads (n = 3) or transvenous leads (n = 2). Of the 2 patients with transvenous pacemakers, 1 patient had an infection that required combined lead extraction and leadless pacemaker implantation;^6^ in the second patient, a leadless pacemaker was implanted without extraction because the patient refused the extraction procedure.^7^ To our knowledge, our case represents the second case of combined extraction and insertion of a leadless pacemaker and the first case of using the Micra device with AV synchrony (VDD mode) after a Senning procedure in d-TGA. Achieving AV synchrony in these patients is very important because it affects systolic and diastolic function as well as overall functional capacity.

## Conclusions

Micra AV2 implantation after lead extraction in a post-Senning d-TGA patient demonstrated effective AV synchrony, representing a viable and innovative approach in complex congenital heart disease management.

**Visual Summary:** Timeline of Case Presentation and Follow-Up

Time	Event/Finding	Key Action/Outcome
Age 11 mo	Senning procedure for d-TGA	Successful atrial switch repair
Age 20 y	Complete AV block	Underwent transvenous dual-chamber pacemaker implantation
Age 30 y	Infected pacemaker with lead protrusion	Decision for extraction + leadless pacemaker
Age 30 y	Transvenous lead extraction + Micra AV2 implantation	AV synchrony achieved (VDD mode)
Postprocedure	Discharge after 24 h	Stable pacing, no complication
Follow-up (1 y)	ECG showing AV-synchronous ventricular pacing (VDD mode)	Stable device parameters

AV = atrioventricular; d-TGA = dextro-transposition of the great arteries; ECG = electrocardiogram.

### Data Availability

The data presented in the manuscript are available upon request to the corresponding author.

## Funding Support and Author Disclosures

The authors have reported that they have no relationships relevant to the contents of this paper to disclose.

## Appendix

Video 1 Contrast Injections in the Right Anterior Oblique Projection Through the Delivery System Demonstrating the Apicoseptal Position of the Micra Device

Video 2 Contrast Injections in the Left Anterior Oblique Projection Through the Delivery System Demonstrating the Apicoseptal Position of the Micra Device
